# Community groups or mobile phone messaging to prevent and control type 2 diabetes and intermediate hyperglycaemia in Bangladesh (DMagic): a cluster-randomised controlled trial

**DOI:** 10.1016/S2213-8587(19)30001-4

**Published:** 2019-03

**Authors:** Edward Fottrell, Naveed Ahmed, Joanna Morrison, Abdul Kuddus, Sanjit Kumer Shaha, Carina King, Hannah Jennings, Kohenour Akter, Tasmin Nahar, Hassan Haghparast-Bidgoli, A K Azad Khan, Anthony Costello, Kishwar Azad

**Affiliations:** a UCL Institute for Global Health, Faculty of Population Health Sciences, University College London, London, UK; b Diabetic Association of Bangladesh, Dhaka, Bangladesh

## Abstract

**Background:**

Strategies are needed to prevent and control type 2 diabetes and intermediate hyperglycaemia, which together affect roughly a third of adults in Bangladesh. We aimed to assess the effects of mHealth and community mobilisation on the prevalence of intermediate hyperglycaemia and diabetes among the general adult population in rural Bangladesh, and to assess the effect of these interventions on the incidence of type 2 diabetes among people with intermediate hyperglycaemia within the study population.

**Methods:**

DMagic was a three-arm, cluster-randomised trial of participatory community mobilisation, mHealth mobile phone messaging, and usual care (control) in 96 villages (population roughly 125 000) in Bangladesh. Community mobilisation involved 18 monthly group meetings, led by lay facilitators, applying a participatory learning and action (PLA) cycle focused on diabetes prevention and control. mHealth involved twice-weekly voice messages over 14 months promoting behaviour change to reduce diabetes risk. The primary outcomes were the combined prevalence of type 2 diabetes and intermediate hyperglycaemia in the overall population at the end of the intervention implementation period, and 2-year cumulative incidence of type 2 diabetes in a cohort with intermediate hyperglycaemia at baseline. Primary outcomes were assessed through fasting blood glucose concentrations and 2-h oral glucose tolerance tests among a cross-section of adults aged 30 years and older and a cohort of individuals identified with intermediate hyperglycaemia. Prevalence findings are based on a cross-sectional survey at the end of the study; incidence findings are based on 2-year follow-up survey of a cohort of individuals identified with intermediate hyperglycaemia through a cross-sectional survey at baseline. We also assessed the cost-effectiveness of the interventions. This trial is registered with the ISRCTN registry, number ISRCTN41083256, and is completed.

**Findings:**

The study took place between June 27, 2015, and June 28, 2018, with the PLA intervention running in 32 villages from June, 2016, to December, 2017, and the mHealth intervention running in 32 villages from Oct 21, 2016, to Dec 24, 2017. End-of study prevalence was assessed in 11 454 individuals and incidence in 2100 individuals. There was a large reduction in the combined prevalence of type 2 diabetes and intermediate hyperglycaemia in the PLA group compared with the control group at the end of the study (adjusted [for stratification, clustering, and wealth] odds ratio [aOR] 0·36 [0·27–0·48]), with an absolute reduction of 20·7% (95% CI 14·6–26·7). Among 2470 adults with intermediate hyperglycaemia at baseline, 2100 (85%) were followed-up at 2 years. The 2-year cumulative incidence of diabetes in this cohort was significantly lower in the PLA group compared with control (aOR 0·39, 0·24–0·65), representing an absolute incidence reduction of 8·7% (3·5–14·0). There was no evidence of effect of mHealth on combined prevalence of intermediate hyperglycaemia and diabetes (aOR 0·93, 0·74–1·16) or the incidence of diabetes (1·02, 0·73–1·43). The incremental cost-effectiveness ratios for PLA were INT$316 per case of intermediate hyperglycaemia or type 2 diabetes prevented and $6518 per case of type 2 diabetes prevented among individuals with intermediate hyperglycaemia.

**Interpretation:**

Our data provide strong evidence to support the use of community mobilisation based on PLA to prevent type 2 diabetes in this rural Bangladeshi population. Despite raising knowledge and awareness of diabetes, the mHealth intervention did not change disease outcomes in our population. Replication studies in other populations should be a priority.

**Funding:**

UK Medical Research Council.

**Research in context**

**Evidence before this study**

We searched PubMed, CINAHL, EMBASE, Web of Science, and Google Scholar for systematic reviews and published original studies on non-pharmacological interventions for the prevention and control of type 2 diabetes published up to July, 2015, with a particular focus on mHealth and community mobilisation interventions in low-income and middle-income countries. We used the search terms “mHealth”, “digital interventions”, “community interventions”, “community groups”, “peer support”, “peer education”, and “community participation” in combination with “diabetes management”, “diabetes prevention”, and “chronic disease”. There were no language restrictions used. Because we wanted to understand both the nature of existing interventions and their effectiveness, we were interested in a range of studies including randomised controlled trials, pilot studies, case-control studies, and qualitative research. Previous studies have shown that mHealth techniques might affect health behaviours, including treatment adherence, weight loss, diet, and exercise, and might reduce the incidence of type 2 diabetes among high-risk individuals. Good evidence exists for group-support and peer-support lifestyle interventions in the prevention or delaying of the onset of type 2 diabetes. Evidence supports the involvement of the community and peer support as a cost-effective means of promoting lifestyle changes in high-income settings, but research in resource-poor settings is lacking. Evidence on the effects of mHealth and community mobilisation on diabetes and related risk factors among the general population, as opposed to high-risk individuals, is yet to emerge from low-income or middle-income countries.

**Added value of this study**

The Bangladesh DMagic trial provides the first large-scale, population-level evidence concerning the effectiveness and cost-effectiveness of mHealth and community mobilisation interventions for reducing the prevalence of intermediate hyperglycaemia and type 2 diabetes and the incidence of type 2 diabetes. Both interventions were acceptable to participants and achieved large population coverage. The mHealth intervention increased knowledge and awareness of type 2 diabetes and its risk factors but had no detectable impact on disease outcomes. Community mobilisation using a participatory learning and action (PLA) approach not only increased knowledge and awareness of disease, but also significantly reduced population prevalence of diabetes and intermediate hyperglycaemia and the incidence of type 2 diabetes among an intermediate hyperglycaemic cohort. National scale-up of PLA in Bangladesh could prevent about 240 000 cases of type 2 diabetes and intermediate hyperglycaemia each year, representing savings in health-care costs of INT$132 million per year.

**Implications of all the available evidence**

The effect size of the PLA community mobilisation on blood glucose is compelling and was robust to sensitivity analysis. Based on the philosophy of Paulo Freire and building on earlier evidence of effectiveness on maternal, neonatal, and child health, ours is the first study to show effectiveness of PLA on risk of type 2 diabetes. The observed effect of facilitated discussion, mutual learning, and collective action is an important challenge to the individualised nature of behavioural interventions, which have shown little success in reducing diabetes risk in general populations. However, as the first study of its kind, replication studies in Bangladesh and elsewhere should be a research priority. The absence of major quantifiable changes in behavioural indicators related to diet, physical activity, and care seeking demands further exploration and hints at complex, ecological mechanisms of action. Lack of evidence of an effect of mHealth on disease outcomes in our general population contrasts with findings from mHealth interventions that target high-risk individuals in other settings. Mixed-methods implementation research will be essential to better understand and develop population-level interventions that stimulate contextually specific actions to prevent and control type 2 diabetes.

## Introduction

The global prevalence of diabetes was estimated to be 9% among adults in 2016 and about 75% of people living with diabetes were in low-income and middle-income countries (LMICs).^1^ Roughly 20–30% of adults in rural areas of Bangladesh have abnormal fasting glucose or impaired glucose tolerance (together termed intermediate hyperglycaemia) and about 10% have diabetes,^2^, ^3^, ^4^ with the prevalence of diabetes (mostly type 2 diabetes) expected to reach 24–34% by 2030.^5^ Despite the large burden of diabetes and intermediate hyperglycaemia in Bangladesh, awareness and knowledge is low^6^ and effective strategies to prevent and control diabetes are urgently needed.

Lifestyle and non-pharmacological interventions can prevent or delay the onset of type 2 diabetes.^7^ Individual targeted strategies that use mobile phone technology (mHealth) have been shown to reduce the incidence of type 2 diabetes in high-risk individuals,^8^ but have not been shown to affect behaviour change and diabetes status among a general, rural population. Community-based and peer support interventions might be a cost-effective means of promoting lifestyle changes in LMICs,^9^, ^10^, ^11^ although a recent trial in India showed no effect on disease outcomes.^12^ Participatory learning and action (PLA) is a specific approach to community mobilisation that engages communities to identify and address their own local problems. It has been shown to improve maternal and newborn survival in LMICs^13^ and might also improve child health^14^, ^15^ and women's reproductive health.^16^

We aimed to separately assess the effects of mHealth health messaging and PLA community mobilisation on the prevalence of intermediate hyperglycaemia and diabetes among the general adult population in rural Bangladesh, and to assess the effect of these interventions on the incidence of diabetes among people with intermediate hyperglycaemia within the study population.

## Methods

### Study design and participants

The DMagic (Diabetes Mellitus: Action through community Groups or mHealth Information for better Control of population blood glucose, risk factors, knowledge and care seeking) trial was done in 96 villages (total population about 125 000) across four selected subdistricts (upazillas) in Faridpur district, Bangladesh, from June 27, 2015 to June 28, 2018. 24 villages with population between 750 and 2500 were selected in each upazilla to minimise contamination via contiguous borders between villages, with buffer, non-study villages separating most clusters. Intervention mapping at the beginning of the study revealed no recent or ongoing community-based programmes specifically designed to reduce the burden of non-communicable diseases, including type 2 diabetes.

DMagic was a three-arm, stratified, cluster-randomised controlled trial in which villages were the units of randomisation and men and non-pregnant women aged 30 years and older were the units of analysis. We did 10 months of formative research and intervention development, including a baseline survey of intermediate hyperglycaemia, diabetes, and non-communicable disease risk factors, and piloting of the PLA intervention; an 18-month intervention phase; and an 8-month post-intervention phase, including an end-of-study survey and analysis. Process evaluation was done concurrently to describe the intervention implementation and explore mechanisms of effect.

The trial received ethical approval from University College London, London, UK (4766/002) and the Diabetic Association of Bangladesh, Dhaka, Bangladesh (BADAS-ERC/EC/t5100246). All survey participants provided informed consent through signature or thumbprint. The study protocol has been previously published.^17^ Data collection, management, and analytical procedures were monitored by an independent data monitoring committee. Trial management was also reviewed by an independent trial steering committee.

### Randomisation and masking

At the outset of the study, we held a public orientation meeting in Faridpur where we obtained community consent and, using stratified randomisation, the 96 villages were randomly allocated (1:1:1) to the mHealth intervention, the community mobilisation (PLA) intervention, or control, with each upazilla constituting one stratum.^17^ The name of each village was written on pieces of paper, colour-coded by upazilla, which when folded were indistinguishable from each other. For each upazilla, the 24 folded pieces of paper were placed in a bottle and then drawn by community leaders and representatives at the public orientation meeting. The first eight villages per upazilla drawn from the bottle were allocated to Arm A, the next eight villages to Arm B, and the final eight villages to Arm C. After all 96 villages had been allocated (32 to each trial arm), each of the three arms were randomly assigned to either the mHealth intervention, community mobilisation (PLA) intervention, or the control group by simultaneous drawing of arm letter and intervention allocation from two separate bottles. Because of the nature of the interventions being tested, the intervention team could not be masked to allocation. The data collection team was masked to allocation at the cluster and individual level during the baseline survey. Primary outcome analysis was done masked to allocation.

### Procedures

The villages allocated to the control group received usual care, which in this context is care seeking in government or private facilities (which is often associated with out-of-pocket payment for blood glucose testing, consultations, and treatments), and little or no preventative public health campaigning.

The mHealth intervention consisted of twice-weekly health behaviour and awareness-raising voice messages sent to participants' mobile phones over a period of 14 months. Message content included information on signs, symptoms, prevention, and care for type 2 diabetes, and provided examples of strategies to reduce the risk of type 2 diabetes and its complications (appendix). Message content was informed by formative research and behaviour change theories,^18^, ^19^ and was reviewed by medical experts. Messages were about 1 min duration and had various formats, including mini-dramas, dialogues, and songs. The intervention was available to anyone with access to a mobile phone in the intervention areas (>95%) who volunteered their mobile phone number to community recruiters at the beginning of the intervention period or at 3 months into the intervention period.

The PLA intervention entailed monthly group meetings, with an average of 27 members per group, led by a lay facilitator who guided participants through a four-phase PLA cycle focused on type 2 diabetes prevention and control. Groups were open to all community members, and people with type 2 diabetes or who were deemed to be at high risk of non-communicable diseases were particularly encouraged to attend. Through the PLA cycle, community members identified behavioural, social, and environmental threats to their health and barriers to healthy lifestyles, prioritised these, and then planned, implemented, and evaluated strategies to address these threats. Awareness raising, exercising in groups, local coordination of blood sugar testing, income generation, and kitchen gardening to increase access to healthy food were popular strategies. Facilitators were locally recruited men and women who had completed higher secondary certificate level of education and were recruited by the study team following assessment of their communication skills, motivation, and familiarity with the study areas. Facilitators received a total of 14 days' training about PLA and community entry, group facilitation, and the basics of type 2 diabetes symptoms, prevention, and control. Each facilitator was responsible for running six to nine PLA groups each month. In addition, an equal number of men's and women's groups were established within each village, with a total of 122 groups facilitated by 16 facilitators (eight men, eight women) across 32 villages. Joint meetings of men's and women's groups were encouraged after phase 1 of the PLA cycle (ie, after identification and prioritisation of health determinants).

Training of informal health workers in the prevention and control of type 2 diabetes was done by the Diabetic Association of Bangladesh across all intervention and control villages during the intervention period. Project mapping of services in the study areas identified all informal care providers (eg, village doctors, pharmacy owners), who were then invited to participate in service-strengthening activities on a voluntary basis. This service strengthening included day-long workshops and provision of guidelines to cadres of largely unregulated care providers who had not received formal accredited training but might have had some degree of informal training through apprentices, workshops, or seminars. These informal care providers are typically the first point of care in health seeking by individuals in rural Bangladesh.^20^

A sampling frame of all permanent residents aged 30 years and older was developed from a household census done between Aug 21, and Oct 28, 2015. 143 households with at least one eligible resident were then selected from each village by use of probability proportional to size sampling. A single eligible adult was selected from each of the 143 households for inclusion in the survey via simple random sampling. A baseline cross-sectional survey among the sampled individuals to obtain sociodemographic characteristics, behaviours, and knowledge of type 2 diabetes was done between Jan 23, and May 30, 2016. The survey included an overnight fasting blood glucose measurement in whole capillary blood obtained by finger prick in the middle or ring finger. All individuals without diagnosed type 2 diabetes then received a 75 g glucose load dissolved in 250 mL water. A 2-h post-prandial repeat capillary blood test was done to determine glucose tolerance status and differentiate between individuals with intermediate hyperglycaemia (defined as impaired fasting glucose or impaired glucose tolerance) and those with type 2 diabetes, based on WHO criteria (appendix).^21^ These baseline data were used to identify an intermediate hyperglycaemia cohort and to compare sociodemographic characteristics between the three trial groups.

Following intervention, the sampling frame was updated and a new random sample of adults aged 30 years and older was selected via the same sampling method as used at baseline. By chance, approximately 25% of individuals sampled for the end-of-study survey had also been included in the baseline survey. In addition, all individuals identified with intermediate hyperglycaemia at baseline were followed-up in the end-of-study survey to measure type 2 diabetes incidence in this cohort. An end-of-study survey of sociodemographic data, knowledge and behaviours, and anthropometric measures of weight, height, blood pressure, and fasting and 2 h post-prandial blood glucose measures was completed in the random cross-sectional sample and the baseline intermediate hyperglycaemia cohort between Jan 16, and April 30, 2018, using the same methods as at baseline.

Data were collected by 16 pairs of fieldworkers (one man and one woman in each pair) with at least secondary education, who underwent extensive training in survey methods, including supervised field practice. Most data collection took place in testing centres established by the field team for the purposes of the study, with additional data collection with pretested questionnaires taking place at respondents' homes.^17^, ^22^ Data collectors were supervised by four field supervisors responsible for observing and verifying data. Data quality-control measures were implemented within the direct digital data capture system used (eg, range and consistency checks), through repeat measures by supervisors on a random basis, and where outlier data were detected on data inspection. Data collectors, supervisors, and managers were unaware of randomisation assignments at baseline, but might have been able to deduce assignment during data collection at the end of the study. Access to end-of-study data was restricted to the monitoring and evaluation managers until collection was complete, at which point the data were available for masked analysis by the lead author (EF).

Process evaluation data will be reported in detail elsewhere. We collected data in all four intervention upazillas of Faridpur district, including small group discussions with men and women attendees, with small groups of men and women with type 2 diabetes, and with group non-attenders. Later we met groups of men and women to explore triangulation and seek consensus on community changes. We also met with men's and women's group facilitators. In some of the groups we used participatory photography where they had identified and represented issues of importance to them using mobile phone cameras. Focus group discussions were digitally recorded and one author (KAk) made notes about the findings in English and translated field observation notes to English for analysis. Key themes around individual, household, and community change were compared with the theory of change drafted after the formative phase of research.

### Outcomes

We prespecified two primary outcomes: the prevalence of intermediate hyperglycaemia and type 2 diabetes at the end of the study and the 2-year cumulative incidence of type 2 diabetes among the cohort with baseline intermediate hyperglycaemia.

Secondary outcomes were mean diastolic and systolic blood pressure, prevalence of hypertension, hypertension control (among those with known hypertension), BMI, prevalence of overweight and obesity and abdominal obesity (waist to hip ratio >0·9 for men and >0·85 for women), health related quality of life (using EQ-5D score), physical activity, fruit and vegetable consumption, and knowledge of the causes, symptoms, complications, prevention and control of type 2 diabetes. Additional secondary outcomes among people with type 2 diabetes were self-awareness of diabetes status and, among those with known diabetes, prevalence of diabetes control, psychological distress (with SRQ-20 screening tool), and receipt of professional medical treatment or advice for diabetes. Full specification of secondary outcomes and methods of assessment have been described previously.^17^

Additional pre-specified outcomes were intervention costs, incremental cost-effectiveness ratios and costs per disability adjusted life years (DALYs) averted for any effective intervention, and process indicators of intervention coverage and qualitative assessments of behaviour change caused by the interventions. An additional outcome of diabetes-only prevalence (excluding intermediate hyperglycaemia) was included post-hoc. Key findings from the qualitative, process assessments of behaviour change are sumarised below, but full analyses are to be reported in a forthcoming publication.

### Statistical analysis

We estimated that a target sample of 143 adults per village (total 13 728, including 10% oversample for non-response) would provide 80% power at 95% confidence to detect a minimum 21·5% reduction in combined prevalence of type 2 diabetes and intermediate hyperglycaemia and 78% power to detect a 33% reduction in cumulative incidence of type 2 diabetes among the baseline intermediate hyperglycaemia cohort in intervention clusters relative to control clusters.^17^

Analysis of intervention effect on the combined prevalence of type 2 diabetes and intermediate hyperglycaemia included all individuals who provided blood glucose measurements in the end-of-study sample survey. This included individuals who provided a random blood glucose measure on the basis of self-reported diagnosis of type 2 diabetes by a medical professional. For the cumulative incidence of type 2 diabetes among the intermediate hyperglycaemia cohort, the analysis population was all individuals for whom a baseline blood glucose measurement revealed intermediate hyperglycaemia and for whom an end-of-study blood glucose measurement was taken.

All analyses were done on an intention-to-treat basis. Comparison between interventions relative to control used random-effects logistic regression, allowing for the stratified and clustered nature of the data. An additive model was used to estimate absolute differences for the primary outcomes. Baseline data were examined by the research team for imbalance between anonymised randomised villages and it was agreed with the data monitoring committee that an apparent marginal difference in baseline household wealth quintiles (derived from principal component analysis of household assets) between study groups would be adjusted for in end-of-study multivariate analyses. Prespecified sensitivity analysis of primary outcomes assessed effects of missing data using multiple imputation and screening effects of individuals being included in the baseline and end-of-study surveys. Post-hoc sensitivity analysis assessed sampling error, enumerator bias, and blood glucose measurement bias (ie, by running analysis on continuous blood glucose measurements and by separately applying different arbitrary fasting blood glucose cut-offs of 5·5 mmol/L, 6·3 mmol/L, and 7·8 mmol/L and 2-h blood glucose cut-offs of 6·8 mmol/L and 10·4 mmol/L for classifications of intermediate hyperglycaemia or diabetes). We also restricted primary outcome analysis to individuals with normal blood glucose levels at baseline who also happened to be included in the end-of-study survey. In view of the high prevalence of intermediate hyperglycaemia and type 2 diabetes and following recommendation from reviewers, we calculated a post-hoc estimate of PLA effect size compared to control as relative risk by use of log binomial models estimated by generalised estimating equations with robust SEs to account for clustering and then calculated the number needed to treat (NNT). Finally, in view of the clinical relevance of type 2 diabetes as an outcome in its own right (ie, not combined with intermediate hyperglycaemia), we did a post-hoc analysis in which we assessed intervention effects on a diabetes-only outcome.

Primary analysis was done by the trial principal investigator (EF), who was masked to treatment allocation and who reported the results to the data monitoring committee and chair of the trial steering committee, after which the identities of the trial groups were revealed, and analysis continued unmasked.

Prespecified secondary outcomes analyses were based on complete data only. Comparative analysis used random-effects logistic regression for binary outcomes and mixed-effects linear regression for continuous outcomes, each allowing for clustering and upazilla stratification. Continuous outcome measures with a skewed distribution were log-transformed before regression analysis. All quantitative analyses were done using STATA/SE version 15.1.

Intervention implementation and coverage was estimated from process evaluation data. Total cost and cost-effectiveness analysis of the DMagic interventions was done initially from a provider (health system) perspective, including costs to the programme provider and public health-care providers (ie, costs associated with increasing service demand and utilisation). Incremental cost-effectiveness ratios were calculated in terms of cost per case of intermediate hyperglycaemia and type 2 diabetes prevented in the general population sample, cost per case of type 2 diabetes prevented among the high-risk cohort with intermediate hyperglycaemia at baseline, and costs per DALY averted. All costs were adjusted for inflation, discounted at 3% per year, and converted to 2018 international dollars (INT$). We calculated DALYs averted using the Global Burden of Disease study approach (appendix);^23^, ^24^ a detailed description of the economic evaluation methodology is presented elsewhere.^25^ Since our interventions and evaluation of them were population based (as opposed to an individual-level study) and, for the most part, we did not follow up the same individuals from baseline to end of study, we were not able to estimate quality-adjusted life years (QALYs) gained.

This trial is registered with the ISRCTN registry, number ISRCTN41083256, and is completed. The trial was registered on March 30, 2016, which was after the initial phase of the study but before any intervention delivery began. In view of the study design, it was not feasible to register the trial before this date, since cluster selection was purposeful (ie, based on population size and non-contiguous borders with other clusters) and identification of clusters was integral to project design and planning.

### Role of the funding source

The funder of the study had no role in study design, data collection, data analysis, data interpretation, or writing the report. The corresponding author had full access to all the data in the study and had final responsibility for the decision to submit for publication.

## Results

All 96 villages agreed to participate and were randomly assigned, 32 to each of the three trial groups. Survey and/or anthropometric baseline data were collected from 12280 (89·5%) of 13 684 individuals between Jan 23, and May 30, 2016. The target sample was slightly smaller than expected because two villages only had 128 and 114 eligible individuals living in separate households. From the baseline survey, 2470 individuals were identified with intermediate hyperglycaemia. Baseline characteristics were similar among all study groups (table 1, appendix), apart from a small difference in household wealth (higher in PLA villages), which was later adjusted for in multivariate analyses.

**Table 1:** Baseline characteristics

		**Control**	**mHealth**	**PLA**
**Cluster level**				
Villages (clusters)		32	32	32
Mean village population aged ≥30 years (SD)		521 (189)	551 (152)	548 (225)
Mean number of households (SD)		269 (97)	282 (79)	285 (112)
**Individual level**				
Total participants who completed survey		4048	4071	4021
Age, years				
	30–39	1391 (34%)	1329 (33%)	1388 (35%)
	40–49	991 (24%)	1068 (26%)	992 (25%)
	50–59	767 (19%)	765 (19%)	761 (19%)
	60–69	648 (16%)	659 (16%)	610 (15%)
	70–100	251 (6%)	250 (6%)	270 (7%)
Sex				
	Men	1950 (48%)	1845 (45%)	1889 (47%)
	Women	2098 (52%)	2226 (55%)	2132 (53%)
Education				
	None	2116 (52%)	1950 (48%)	1905 (47%)
	Primary	921 (23%)	852 (21%)	1004 (25%)
	Secondary	989 (24%)	1231 (30%)	1086 (27%)
	Tertiary	22 (1%)	38 (1%)	26 (1%)
Literacy				
	Literate	1455 (36%)	1649 (41%)	1561 (39%)
	Illiterate	2593 (64%)	2422 (59%)	2460 (61%)
Marital status				
	Married	3528 (87%)	3574 (88%)	3530 (88%)
	Not married	520 (13%)	497 (12%)	491 (12%)
Religion				
	Muslim	3660 (90%)	3674 (90%)	3666 (91%)
	Other	388 (10%)	397 (10%)	355 (9%)
Occupation				
	Not working	2216 (55%)	2323 (57%)	2164 (54%)
	Manual labour	1362 (34%)	1296 (32%)	1376 (34%)
	Non-manual labour	468 (12%)	452 (11%)	481 (12%)
	Missing data	2 (<1%)	0	0
Wealth quintile				
	Most poor	845 (21%)	910 (22%)	676 (17%)
	Very poor	781 (19%)	896 (22%)	768 (19%)
	Poor	822 (20%)	821 (20%)	798 (20%)
	Less poor	855 (21%)	722 (18%)	826 (21%)
	Least poor	745 (18%)	722 (18%)	953 (24%)
Total participants with baseline data for glycaemic status*		4070	4063	4054
Glycaemic status				
	Normal	2860 (70%)	2816 (69%)	2801 (69%)
	Impaired fasting glucose†	200 (5%)	186 (5%)	203 (5%)
	Impaired glucose tolerance†	632 (16%)	655 (16%)	594 (15%)
	Diabetes‡	378 (9%)	406 (10%)	456 (11%)

PLA=participatory learning and action.

* Glycaemic status missing for 38 participants in the control group, 30 participants in the mHealth group, and 25 participants in the PLA group (excluded from totals).

† Intermediate hyperglycaemia was defined as impaired fasting glucose or impaired glucose tolerance.

‡ Based on blood glucose measurement or self-reported previous medical diagnosis.^21^

**Figure 1:**
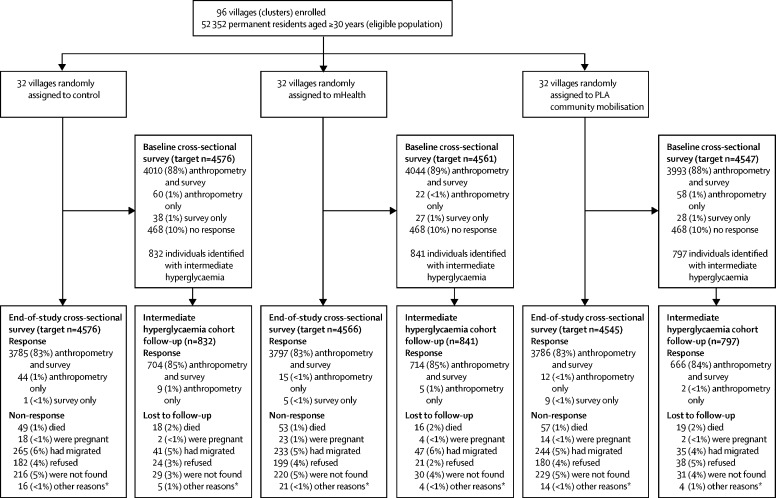
Trial profile Anthropometry includes all physical measures—ie, blood glucose, blood pressure, weight, height, and waist and hip circumferences. PLA=participatory learning and action. *Other reasons were physical or mental disability or severe illness preventing participation, incarceration, or temporary residence only.

End-of-study data were collected from 11 454 (83·7%) of 13 687 individuals between Jan 16, and April 30, 2018. The end-of-study cross-sectional sample was similar in terms of sociodemographic characteristics to the baseline sample and between study groups (appendix). Of the 2470 individuals with intermediate hyperglycaemia at baseline, 2100 were followed-up (85%; figure 1). Non-responders were more likely to be men (1712 [23%] of 7520 men *vs* 721 [9%] of 7854 women; p<0·0001; figure 1) and men non-responders were younger than men responders (mean difference 2·0 years; p<0·0001), whereas women non-responders were slightly older than women responders (mean difference 3·1 years; p<0·0001). The same patterns of non-response were seen across all study groups.

Exposure to the interventions was fairly high (table 2). In the mHealth intervention group, 2613 (61%) of 4320 villagers reported ever receiving a mobile phone message; in the PLA group, 3136 (74%) of 4247 reported ever participating in a group.

**Table 2:** Intervention coverage process indicators

	**PLA (n=4247)**	**mHealth (n=4320)**	**Control (n=4292)**
Ever participated in PLA community group	3136 (74%)	1 (<1%)	1 (<1%)
Knows someone who attended PLA community group	3046 (72%)	10 (<1%)	0 (<1%)
Ever received mHealth message	55 (1%)	2613 (60%)	7 (<1%)
Knows someone who received mHealth messages	32 (1%)	2845 (66%)	6 (<1%)

Data are n (%). PLA=participatory learning and action.

**Figure 2:**
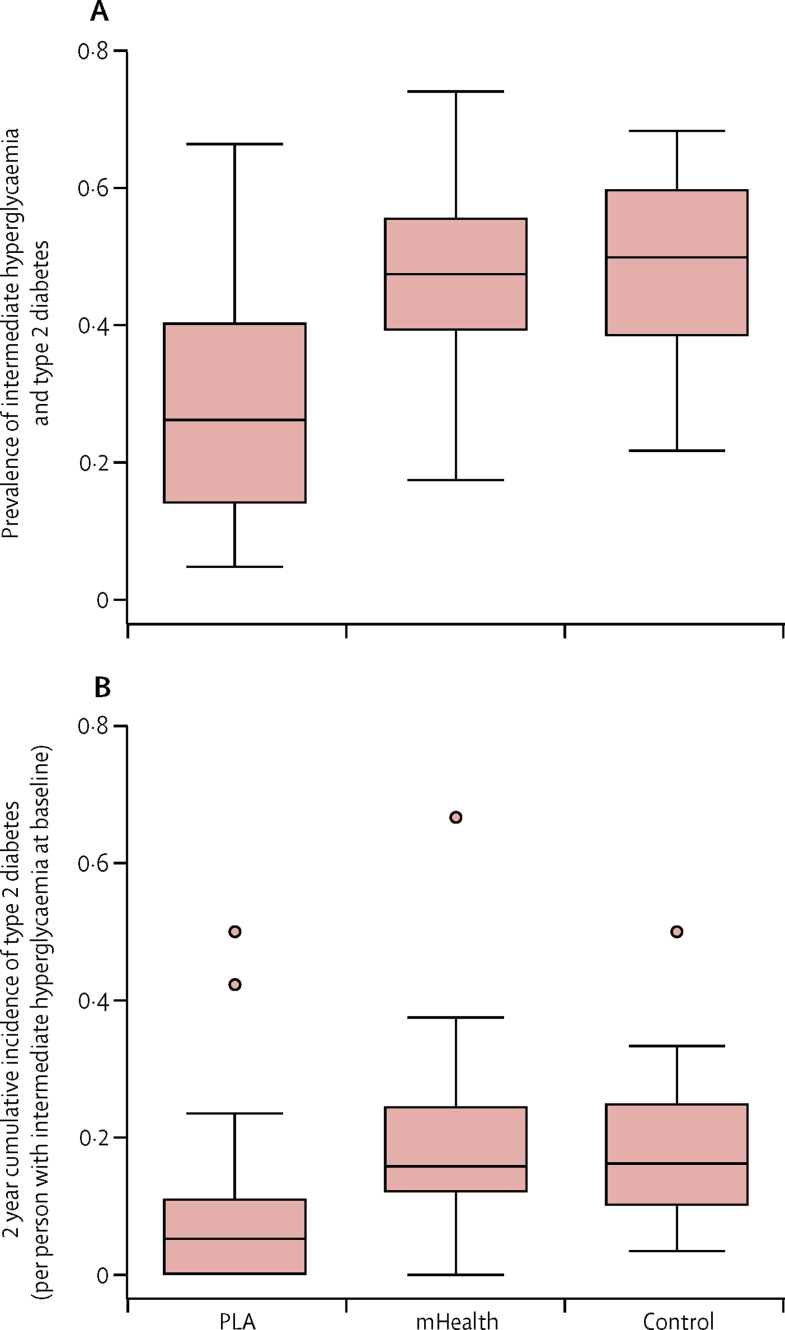
Allocation box-plots showing (A) a cluster-level summary of intermediate hyperglycaemia and diabetes at end of study and (B) cluster-level 2 year cumulative incidence of diabetes among the hyperglycaemic cohort Box-plot shows median, minimum and maximum values and interquartile range.

At the end-of-study survey, the combined prevalence of type 2 diabetes and intermediate hyperglycaemia was significantly lower in the PLA group compared with the control group (table 3, figure 2). The 2-year cumulative incidence of type 2 diabetes among the intermediate hyperglycaemia cohort in the PLA group compared with the control group was also significantly reduced (table 3, figure 2). Our post-hoc analysis of the effect on type 2 diabetes prevalence only (ie, not combined with intermediate hyperglycaemia) showed that the PLA intervention reduced the prevalence of type 2 diabetes by 48% relative to control (305 [8%] of 3757 *vs* 493 [13%] of 3821; adjusted [stratified, clustered design, and wealth] odds ratio [aOR] 0·52, 0·38–0·71; p<0·0001). There was no evidence of an effect of the mHealth intervention on the combined prevalence of type 2 diabetes and intermediate hyperglycaemia or the incidence of type 2 diabetes in the intermediate hyperglycaemia cohort compared with control villages (table 3, figure 2). There was also no evidence of an effect of the mHealth intervention on the prevalence of type 2 diabetes alone in our post-hoc analysis (563 [15%] of 3797 *vs* 493 [13%] of 3821; adjusted [stratified, clustered design, and wealth] aOR 1·18, 0·95–1·48; p=0·139).

**Table 3:** Primary outcome measures at end of study

		**Control**	**mHealth**	**PLA**
Population prevalence of intermediate hyperglycaemia and diabetes*†				
Total population		3821	3797	3757
Normoglycaemic		1960 (51·3%)	2003 (52·8%)	2686 (71·5%)
Diabetes or intermediate hyperglycaemic		1861 (48·7%)	1794 (47·2%)	1071 (28·5%)
Relative difference (odds ratio [95% CI])				
	Unadjusted (allowing for stratified clustered design)	Reference	0·94 (0·75–1·18; p=0·611)	0·37 (0·27–0·49; p<0·0001)
	Adjusted for household wealth quintile and allowing for stratified clustered design	Reference	0·93 (0·74–1·16; p=0·513)	0·36 (0·27–0·48; p<0·0001)
Absolute risk difference, % points (95% CI)				
	Unadjusted (allowing for stratified clustered design	Reference	–1·4 (−6·9 to 4·1; p=0·626)	–20·1 (−26·1 to −14·0; p<0·0001)
	Adjusted for household wealth quintile and allowing for stratified clustered design	Reference	–1·7 (−7·3 to 3·9; p=0·542)	–20·7 (−26·7 to −14·6; p<0·0001)
**2 year cumulative incidence among intermediate hyperglycaemic cohort**†				
Total population		712	717	665
Normoglycaemic		249 (35·0%)	280 (39·1%)	407 (61·2%)
Intermediate hyperglycaemic		337 (47·3%)	315 (43·9%)	199 (29·9%)
Diabetes		126 (17·7%)	122 (17·0%)	59 (8·9%)
Relative difference (odds ratio [95% CI])				
	Unadjusted (allowing for stratified clustered design)	Reference	0·99 (0·70–1·39; p=0·941)	0·41 (0·24–0·68; p=0·0005)
	Adjusted for household wealth quintile and allowing for stratified clustered design	Reference	1·02 (0·73–1·43; p=0·912)	0·39 (0·24–0·65; p=0·0003)
Absolute risk difference, % points (95% CI)				
	Unadjusted (allowing for stratified clustered design)	Reference	–0·04 (−5·3 to 5·3; p=0·987)	–8·4 (−13·8 to −3·0; p=0·0023)
	Adjusted for household wealth quintile and allowing for stratified clustered design	Reference	0·36 (−4·7 to 5·5; p=0·889)	–8·7 (−14·0 to −3·5; p=0·0011)

Data are n or n (%), unless otherwise indicated. All p-value comparisons are versus control. PLA=participatory learning and action.

* Coefficient of variation for diabetes and intermediate hyperglycaemia is 0·346.

† Anthropometry participants with missing blood glucose data: eight in the control group, 15 in the mHealth group, and 41 in the PLA group.

‡ Anthropometry participants with missing blood glucose data: one in the control group, two in the mHealth group, and three in the PLA group.

Increases in ability to report one or more valid causes, symptoms, complications, and strategies for prevention and control of diabetes were observed in both intervention groups compared with control, with the effect consistently greatest in the PLA group (table 4). Self-awareness of diabetes status among individuals identified as having type 2 diabetes by blood glucose testing was five-times higher in the PLA group than in the control group (aOR 5·09, 2·95–8·79; p<0·0001), but the smaller increase in the mHealth group compared with control was not significant (aOR 1·44, 0·94–2·19; p=0·092). Improvements in diabetes control among people with known diabetes in the intervention groups compared with control were not significant and there was no intervention effect on receipt of professional care for diabetes among those aware of their disease (table 4).

**Table 4:** Secondary outcome measures at end of study

	**Study group**			Unadjusted coefficient or odds ratio*		**Adjusted coefficient or odds ratio**†	
	PLA (n=3798)	mHealth (n=3812)	Control (n=3829)	PLA vs control	mHealth vs control	PLA vs control	mHealth vs control
Mean diastolic blood pressure (mm Hg)	73·8 (11·2)	72·9 (11·2)	73·9 (11·2)	–0·25 (−1·78 to 1·28; p=0·748)	–1·12 (−2·59 to 0·35; p=0·135)	–0·50 (−2·05 to 1·05; p=0·526)	–1·18 (−2·58 to 0·22; p=0·098)
Mean systolic blood pressure (mm Hg)	125·3 (19·6)	124·4 (20·0)	125·5 (20·8)	–0·19 (−2·54 to 2·17; p=0·877)	–1·05 (−3·46 to 1·35; p=0·391)	–0·53 (−2·90 to 1·83; p=0·661)	–1·10 (−3·44 to 1·25; p=0·360)
Hypertension	958 (25%)	854 (22%)	899 (23%)	1·08 (0·86 to 1·35; p=0·516)	0·94 (0·78 to 1·14; p=0·517)	1·02 (0·81 to 1·29; p=0·837)	0·93 (0·77 to 1·14; p=0·483)
Hypertension control (among those with known hypertension)	131/332 (39%)	122/313 (39%)	90/258 (35%)	1·21 (0·79 to 1·87; p=0·375)	1·12 (0·79 to 1·59; p=0·523)	1·21 (0·78 to 1·87; p=0·389)	1·13 (0·80 to 1·60; p=0·501)
Mean BMI (kg/m^2^)	22·0 (3·6)	21·9 (3·6)	21·9 (3·6)	0·08 (−0·21 to 0·38; p=0·572)	–0·05 (−0·28 to 0·17; p=0·631)	–0·09 (−0·39 to 0·20; p=0·543)	–0·09 (−0·29 to 0·12; p=0·410)
Overweight or obese (waist:hip ratio >0·9 for men and >0·85 for women)	1341 (35%)	1265 (33%)	1326 (35%)	1·03 (0·88 to 1·20; p=0·720)	0·94 (0·83 to 1·06; p=0·286)	0·94 (0·80 to 1·11; p=0·459)	0·92 (0·82 to 1·03; p=0·151)
Abdominal obesity	1964 (52%)	1952 (51%)	1922 (50%)	1·05 (0·74 to 1·48; p=0·791)	1·03 (0·73 to 1·46; p=0·851)	0·98 (0·70 to 1·37; p=0·914)	1·02 (0·72 to 1·44; p=0·916)
Median EQ-5D‡§	0·80 (0·73–1·0)	0·85 (0·73–1·0)	0·80 (0·73–1·0)	0·03 (−0·02 to 0·08; p=0·253)	–0·01 (−0·06 to 0·04; p=0·687)	0·03 (−0·02 to 0·07; p=0·261)	–0·02 (−0·06 to 0·03; p=0·471)
Mean self-rated health§	77·9 (15·5)	76·7 (16·0)	74·5 (16·1)	3·28 (0·37 to 6·19; p=0·027)	2·12 (−0·78 to 5·02; p=0·153)	2·76 (−0·16 to 5·68; p=0·064)	1·96 (−0·97 to 4·88; p=0·189)
Median SRQ-20 score among adults aged ≥30 years with self-reported diabetes	8 (5–12); n=137	6 (4–9); n=144	8 (5–13); n=94	–0·004 (−0·20 to 0·19; p=0·971)	–0·17 (−0·36 to 0·02; p=0·085)	0·006 (−0·19 to 0·20; p=0·949)	–0·16 (−0·35 to 0·03; p=0·105)
Ability to report one or more valid cause of diabetes§	3646 (96%)	2981 (78%)	2153 (57%)	36·7 (18·2 to 73·7; p<0·0001)	3·72 (2·06 to 6·73; p<0·0001)	35·7 (17·7 to 71·9; p<0·0001)	3·77 (2·05 to 6·91; p<0·0001)
Ability to report one or more valid symptom of diabetes§	3659 (96%)	3205 (84%)	2452 (65%)	25·1 (11·8 to 53·2; p<0·0001)	4·34 (2·07 to 9·10; p=0·0001)	24·0 (11·3 to 50·9; p<0·0001)	4·37 (2·07 to 9·24; p=0·0001)
Ability to report one or more valid complication of diabetes§	3649 (96%)	3084 (81%)	2161 (57%)	36·5 (18·5 to 72·0; p<0·0001)	5·29 (2·58 to 10·9; p<0·0001)	35·4 (17·8 to 70·4; p<0·0001)	5·42 (2·60 to 11·3; p<0·0001)
Ability to recognise one or more valid complication of diabetes when prompted§	3723 (98%)	3367 (89%)	2932 (77%)	19·7 (8·24 to 46·9; p<0·0001)	3·87 (1·49 to 10·1; p=0·0056)	18·3 (7·66 to 43·9; p<0·0001)	3·88 (1·47 to 10·2; p=0·0063)
Ability to report one or more valid way to prevent diabetes§	3608 (95%)	3294 (87%)	2626 (69%)	10·6 (5·74 to 19·4; p<0·0001)	4·28 (2·10 to 8·68; p=0·0001)	10·0 (5·44 to 18·5; p<0·0001)	4·31 (2·10 to 8·85; p=0·0001)
Ability to report one or more valid way to control diabetes§	3619 (95%)	3354 (88%)	2796 (74%)	8·81 (4·64 to 16·7; p<0·0001)	3·94 (1·92 to 8·08; p=0·0002)	8·36 (4·42 to 15·8; p<0·0001)	3·93 (1·90 to 8·12; p=0·0002)
Diabetes control among those with known of diabetes	68/123 (55%)	57/134 (43%)	33/86 (38%)	2·17 (0·97 to 4·87; p=0·060)	1·22 (0·51 to 2·94; p=0·657)	2·24 (0·97 to 5·16; p=0·058)	1·21 (0·50 to 2·92; p=0·666)
Self-reported awareness of diabetes status among all those identified as having diabetes by objective blood glucose test	143/342 (42%)	156/652 (24%)	100/580 (17%)	5·10 (2·97 to 8·76; p<0·0001)	1·42 (0·91 to 2·20; p=0·119)	5·09 (2·95 to 8·79; p<0·0001)	1·44 (0·94 to 2·19; p=0·092)
Receipt of professional treatment or advice for diabetes among those with diabetes and aware of their status	114/143 (80%)	128/156 (82%)	84/100 (84%)	0·85 (0·35 to 2·02; p=0·708)	0·97 (0·43 to 2·19; p=0·947)	0·78 (0·32 to 1·92; p=0·584)	0·93 (0·41 to 2·13; p=0·873)
Average ≥150 min physical activity per week§	2844 (75%)	2891 (76%)	2923 (77%)	0·84 (0·54 to 1·30; p=0·435)	1·00 (0·63 to 1·59; p=0·993)	0·83 (0·53 to 1·30; p=0·418)	0·98 (0·62 to 1·57; p=0·945)
Mean number of portions of fruit and/ or vegetables consumed per day¶	4·0 (2·3)	3·4 (1·6)	3·6 (1·6)	0·35 (−0·11 to 0·81; p=0·133)	–0·18 (−0·54 to 0·18; p=0·335)	0·29 (−0·10 to 0·69; p=0·143)	–0·19 (−0·53 to 0·15; p=0·274)

Data are mean (SD), median (IQR), n/N (%), or n (%), unless otherwise indicated. Measures of effect are beta coefficients where the measure is continuous (eg, mean or median) and are odds ratios where the measure is %. PLA=participatory learning and action. SRQ-20=Self-Reporting Questionnaire 20-Item.

* Accounting for stratified clustered design.

† Adjusted for household wealth quintile and accounting for stratified, clustered design.

‡ EuroQol-5D (EQ-5D) using UK tariffs.

§ Survey totals PLA=3795; mHealth=3802; and control=3786.

¶ Survey totals PLA=3792; mHealth=3802; control=3777.

There was no evidence of an effect of either intervention on blood pressure, overweight and obesity, or self-reported physical activity or fruit and vegetable consumption (table 4). Overall quality-of-life score did not differ between study groups, and the crude significant difference in self-rated health measured on a scale of 0 (worst health) to 100 (best health) between the PLA and control groups was attenuated and non-significant when adjusted for household wealth (table 4).

Prespecified sensitivity analyses including adjustment for a possible screening effect among individuals included in the baseline and end-of-study surveys and multilevel multiple imputations for missing blood glucose measurements had negligible effect on the primary analyses (appendix).

In view of the findings for the PLA intervention, we did additional post-hoc sensitivity analyses. First, we assessed potential sampling error and imbalances in key sociodemographic characteristics between study groups by running a predictive regression of our primary outcomes on sociodemographic characteristics at baseline and then checked whether the significant variables in this predictive regression differed between treatment groups. Apart from wealth quintiles, which we had prespecified as a covariate in our model, no other sociodemographic measures were significantly different between treatment groups, lending support to our primary findings (data not shown) and suggesting that our random sampling approach was effective and consistent across study groups.

We then explored potential blood glucose measurement biases and errors. We checked the effect of PLA on the continuous fasting and 2-h blood glucose measures, which showed significant reductions in mean population glucose compared with control (appendix). We also ran a series of regression models on the primary outcomes using different, arbitrary cut-off points for categorisation of intermediate hyperglycaemia and type 2 diabetes; again, our finding of a strong association of reduced prevalence and incidence with PLA exposure remained irrespective of cut-off values used (data not shown). Finally, to rule out potential enumerator effects (ie, potential bias in fieldworker measurements), we included enumerator identifiers as fixed effects in our regression of intervention exposure and fasting and 2 h blood glucose, treated as continuous outcomes. By doing so were able to show that our findings were robust to the inclusion of enumerator fixed effects (appendix) and that the enumerator fixed effects were statistically null (fasting blood glucose enumerator F-test=0·002, p=0·9642; 2 h blood glucose enumerator F-test=0·966, p=0·3257).

We also restricted our incidence analysis to 1457 individuals with normoglycaemic blood glucose measurements at baseline who happened to also be included in our end-of-study sample. Our random-effects regression findings showed a 72% reduction in the combined incidence of intermediate hyperglycaemia and type 2 diabetes among normoglycaemic individuals in PLA villages relative to control (OR 0·28, 0·19–0·42; p<0·0001), suggesting that PLA was also effective among a normoglycaemic population.

For the combined prevalence outcome, the relative risk for PLA compared with control was 0·61 (95% CI 0·50–0·73), with an NNT of 4·75 (95% CI 3·71–6·61). For the 2 year cumulative incidence outcome, the relative risk was 0·49 (0·31–0·80), with an NNT of 10·68 (7·02–22·32).

Both interventions were delivered as per our published protocol, although the mHealth intervention started about 4 months later than originally planned and thus ran for just 14 months (instead of 18 months) due to technical and bureaucratic delays in establishing a system to deliver messages. The 122 PLA groups ran from June, 2016, to December, 2017, with a population coverage of one group per 342 people. In the PLA group, 3136 (74%) of 4247 people in the end-of-study survey population reported participating in group meetings (table 2). Repeat attendance at groups was very high, with about 90% of attenders participating in multiple meetings (data not shown). Roughly 9000 individuals (22% of the total population of around 41 667) provided their mobile phone numbers to receive the mHealth intervention and messages were delivered to about 7400 (82%) of these (around 51% of eligible adults). A total of 120 different messages were developed and sent between Oct 21, 2016 and Dec 24, 2017. An average of 60% of the first 26 messages were received; thereafter we improved the delivery systems, which increased receipt to 86% for the remaining 94 messages. More than 50% of 100 randomly selected message recipients who participated in a process evaluation survey reported sharing messages with others and two-thirds of the end-of-study population in mHealth villages received or knew someone who received the messages (table 2). Contamination between trial groups was minimal.

Details of our qualitative findings will be reported elsewhere. In summary, these data suggest that changes occurred at the individual, household, and community levels in response to the PLA intervention. As awareness of how to prevent type 2 diabetes increased, and individuals were motivated to change their diets, reporting reduced rice, oil, sugar, and salt consumption and increased consumption of a variety of vegetables. Kitchen gardens were encouraged by groups and increased access to vegetables. Both attenders and non-attenders reported an increase in intentional physical activity in PLA intervention areas, particularly among women, as it became more acceptable for them to walk in groups to prevent or control diabetes. Formative data show that few women felt comfortable to walk outside their households before either intervention. In our end-of-study survey, the median self-reported time spent doing physical activity each week was 60 min greater in PLA clusters than in control clusters (ratio of geometric mean of activity time 1·12 [95% CI 0·88–1·43]), although this difference was not significant when we accounted for the stratified, clustered survey design. When men and women from the same household participated in groups, behaviour change was easier to initiate and sustain. PLA groups and their activities made community members feel in control of their health and able to prevent diabetes. The PLA intervention also destigmatised blood glucose testing and healthy behaviours such as physical activity, reduced or no sugar in tea, and healthy eating while socialising.

Total and average annual costs of the PLA intervention were INT$601 484 and $240 594, respectively. Total and average annual costs of the mHealth intervention were $312 630 and $125 052, respectively. The average annual costs of the PLA and mHealth per beneficiary (adults ≥30 years) covered were $14 and $7, respectively, with costs per total population (all ages) being $6 and $3, respectively. The incremental cost-effectiveness ratios for PLA were $316 per case of intermediate hyperglycaemia or type 2 diabetes prevented (or $124 per DALY averted) and $6518 per case of type 2 diabetes prevented (or $2551 per DALY averted) among individuals with intermediate hyperglycaemia at baseline.

## Discussion

We assessed two community interventions to prevent and control type 2 diabetes and intermediate hyperglycaemia in rural Bangladesh. Facilitated PLA community mobilisation led to large, significant reductions in the combined prevalence of type 2 diabetes and intermediate hyperglycaemia and 2-year incidence of type 2 diabetes among an intermediate hyperglycaemia cohort. The mHealth intervention had no effect on diabetes status. Both interventions were associated with improvements in diabetes knowledge, but had no apparent impact on blood pressure, overweight and obesity, or on recalled fruit and vegetable consumption or physical activity.

The effect size of the PLA community mobilisation on blood glucose is surprising, especially in the absence of major quantifiable changes in behavioural indicators related to diet, physical activity, and care seeking. We therefore did several additional data checks and sensitivity analyses to identify alternative explanations for the observed effects. However, our results are robust to examinations for sampling errors, enumerator effects, measurement biases, and response bias. Furthermore, the observed effectiveness of PLA on mean population blood glucose measures suggests that our findings are not an artefact of blood glucose cut-off values used. The effectiveness of the intervention on normoglycaemic individuals adds to the evidence of PLA effectiveness in the general population and not only high-risk individuals. Overall, therefore, our findings are compelling, but replication is needed in other populations in Bangladesh and elsewhere.

Although very different in terms of mode of delivery and, ultimately, impact, there was substantial overlap in terms of the content and focus of our mHealth and PLA interventions. Our findings contribute to existing literature on mHealth interventions in LMICs. Despite potential for relatively low-cost scalability,^26^ mHealth interventions have often been criticised for a lack of robust assessment in terms of health outcomes.^27^, ^28^ Our twice-weekly voice message intervention had a strong basis in behaviour change theory, high population coverage (albeit lower than the PLA intervention), and important impacts on knowledge and awareness of type 2 diabetes, but did not change disease outcomes at the population level. Our findings on mHealth effectiveness differ from those reported by Ramachandran and colleagues,^8^ who noted significant reductions in the incidence of type 2 diabetes among urban working men with impaired glucose tolerance who received prescribed lifestyle changes followed by tailored text messages in southeast India. Our findings also differ to those reported by Islam and colleagues,^29^ who observed improved glycaemic control among patients with type 2 diabetes randomly assigned to receive mobile text messages over a 6-month period.^29^ Although details of the intervention and trial design of Islam and colleagues' study are unclear, differences in population demographics of Ramachandran and colleagues' and Islam and colleagues' trials, the individual-level randomisation, and delivery of text messages to individuals receiving care are notable between these studies and ours. It is also possible that the delayed start, slightly shorter-than-planned mHealth delivery period, and temporary problems in delivering messages to about 40% of registered mobiles at the beginning of the study might have reduced the effectiveness of our intervention.

That disease outcome changes were only apparent in the PLA group suggests wider benefits of participatory interventions beyond the provision of information and modelled behaviour employed in our mHealth intervention. This finding builds on existing evidence of the effectiveness of PLA on maternal, neonatal, and child health,^13^ and ours is the first assessment of this method for a non-communicable disease. Our intervention differs from the recent peer-support lifestyle intervention in Kerala, which improved physical activity and dietary practices but did not affect the incidence of type 2 diabetes among a high-risk cohort.^12^ First, unlike our measurement of population-level effects, the Kerala outcomes were only measured among the high-risk individuals who, in the intervention group, had been assigned to group support. It is possible that our intervention improved certain secondary outcomes such as diet and physical activity among high-risk sub-groups that are not apparent in general population measurement from only survey questions. Second, the peer-support intervention seems to be education-focused and targeted to a high-risk cohort, unlike our inclusive PLA approach. Finally, better education and literacy indices among the Kerala study population notwithstanding, in the patriarchal context of Bangladesh we implemented separate groups for men and women, whereas the Kerala study implemented mixed-sex groups, which might have affected their effectiveness.

Our study has several limitations. Although based on WHO STEPS and the Demographic and Health Survey instruments previously applied in Bangladesh and pilot tested before any data collection, large parts of our survey tools, such as for knowledge of diabetes, were developed for this study and were not formally validated in our study population. Our measure of physical activity was crude and based on recall and analysed as a binary measure of at least 150 min per week; this approach might have missed significant changes in the intensity of population exercise between groups. We did not do a detailed food consumption survey, allowing detailed assessment of changes in consumption of certain foods, such as sugar-sweetened beverages. The large effects of PLA on disease without apparent changes in common diabetes risk factors suggests a complex mechanism of action. Perhaps several small changes in lifestyle and diet combined to produce a strong cumulative effect on blood sugar. Dietary and physical activity behaviour change was self-reported in process evaluation data, which showed that rice, oil, salt, and sugar consumption decreased in PLA villages. Process data suggested that intentional exercise increased, particularly among women. One striking effect of the groups that emerged through process evaluation was that women were able to negotiate time for group exercise in communities where individual women were stigmatised if they walked alone. Messages alone might not be able to change this behaviour.^30^ Furthermore, the removal of stigma about diabetes, the increased solidarity among villagers, and the sharing of information and ideas might have reduced stress levels in the population, which might have contributed to the PLA intervention effect. The differences between process and survey data require further research. The tools we used to measure food consumption and exercise might be too blunt, and participants might be able to more accurately estimate their time spent exercising and quantities of food eaten after the intervention given the clear messaging and emphasis on these behaviours.

Our approach and some of the assumptions we used for calculating DALYs gives a conservative estimate of the total DALYs averted by PLA. The disability weight that we used, though very small, is for uncomplicated type 2 diabetes. We used the same weight for both type 2 diabetes and intermediate hyperglycaemia, which might slightly overestimate the years lived with disability. However, we did not take into account the likelihood that some of the individuals with type 2 diabetes will develop diabetes-related complications, for which disability weights can range from 0·004 to 0·631.^31^ Additionally, since we did not have the exact age of diagnosis or onset of diabetes, we used average age of individuals with diagnosed diabetes in our end-of-study survey as age of onset (or diagnosis) of diabetes. Using this age is likely to underestimate years lived with disability and years of life lost, since many individuals were diagnosed before this age.

As previously noted, our population-level evaluation means we are unable to estimate QALYs gained. However, the cost-effectiveness ratios of INT$316 to $6518 per case of intermediate hyperglycaemia or type 2 diabetes prevented (or $124 to $2551 per DALY averted) suggests that PLA is highly cost-effective according to the WHO cost-effectiveness threshold,^32^ considering Bangladesh's gross domestic product per person of $3869 (in 2017). Scale-up of PLA at the national level could prevent about 240 000 cases of type 2 diabetes or intermediate hyperglycaemia each year (equivalent to about $132 million savings in health-care costs per year), assuming a 30% loss in effect through scale-up (appendix). In the absence of prepayment systems in Bangladesh, most health-care expenditures are incurred by patients and their households, potentially leading to financial catastrophes for many.^33^ Preliminary analyses of our baseline survey data on cost of seeking care suggests that about 60% of people with type 2 diabetes might be at risk of catastrophic and impoverishing health expenditure (ie, spend more than 10% of their household income on diabetes care). Therefore, scaling up cost-effective interventions such as the DMagic PLA has potential to reduce the incidence of impoverishing health expenditure.

The strengths of our study are the large, rural, population-based surveys with high response rates at baseline and the end of the study, and the high 2-year follow-up of the intermediate hyperglycaemia cohort, and assessment of glycaemic status through fasting and 2 h blood glucose tests. Randomisation should eliminate issues of confounding, although baseline differences in wealth were apparent and therefore controlled for, but the possibility of additional unmeasured imbalances between study groups cannot be excluded. Enumerator and participant masking to intervention exposure was infeasible in the end-of-study survey, although the extent to which this could affect the primary outcome measures is likely to be small and we identified no enumerator bias in our sensitivity analysis. Both interventions had good coverage and there was minimal contamination between groups. Although capillary blood glucose concentrations overestimate blood sugar compared with venous samples, the method is feasible and acceptable for epidemiological studies and would not affect the differences we identified between study intervention groups.

Further analysis of process evaluation data is likely to provide a more nuanced understanding of intervention mechanisms and effects. Nevertheless, the large, cost-effective impact of PLA in this trial suggests that it might be beneficial in other LMICs with a high burden of type 2 diabetes, and perhaps among high-risk groups in high-income settings. Replication in other populations is an important next step and follow-up of the DMagic study population with mixed-methods approaches will be important to better explain intervention mechanisms of action and long-term impacts.

## Data sharing

De-identified data collected for this study and a data dictionary are available from the corresponding author on reasonable request.
