# Supplementary material for: Community groups or mobile phone messaging to prevent and control type 2 diabetes and intermediate hyperglycaemia in Bangladesh (DMagic): a cluster-randomised controlled trial
Source: Lancet Diabetes Endocrinol. 2019 Mar;7(3):200–12. doi: 10.1016/S2213-8587(19)30001-4 (PMC6381080; doi:10.1016/S2213-8587(19)30001-4)
Supplement: Supplementary appendix [file mmc1.pdf]

THE LANCET
Diabetes & Endocrinology

Supplementary appendix

This appendix formed part of the original submission and has been peer reviewed. We post it as supplied by the authors.

Supplement to: Fottrell E, Ahmed N, Morrison J, Kuddus A, Shaha S K, King C, et al. Community groups or mobile phone messaging to prevent and control type 2 diabetes and intermediate hyperglycaemia in Bangladesh (DMagic): a cluster-randomised controlled trial. *Lancet Diabetes Endocrinol* 2019; published online Feb 4. http://dx.doi.org/10.1016/S2213-8587(19)30001-4.

Supplementary Methods 1: Estimating the impact of the community mobilisation intervention at scale

We estimated the impact of the community mobilisation intervention if it was rolled out to the whole population of Bangladesh (both rural and urban). The impact was estimated in terms of number of cases of type 2 diabetes and intermediate hyperglycaemia prevented. For estimating the impact at scale, we needed risk ratios (RRs) for the intervention and incidence of diabetes and intermediate hyperglycaemia in Bangladesh.

Risk ratios

We used RR of the intervention for combined type 2 diabetes and intermediate hyperglycaemia (RR 0.61). Also, as a lower bound estimate, we assumed a 30% loss of effectiveness at scale (RR 0.73).

Incidence of diabetes and intermediate hyperglycaemia

We obtained the incidence rate for diabetes from the 2016 Global Burden Diseases study (http://ghdx.healthdata.org/gbd-2016). We assumed a similar incidence rate for intermediate hyperglycaemia.

Saving in health-care costs

We estimated the health-care costs saved by scaling up the intervention at national level, using the impact results at scale. For estimating the saving in health-care costs, we needed average (unit) cost of seeking care for people with type 2 diabetes and intermediate hyperglycaemia. For type 2 diabetes, we used average cost of seeking care (around INT$638, including both direct medical and direct non-medical [such as transportation] costs) from our end-of-study survey; for intermediate hyperglycaemia, we used an assumption of 80% of type 2 diabetes costs, based on the study by Roberts and colleagues.[1] We calculated a weighted average unit cost using average costs for type 2 diabetes and intermediate hyperglycaemia and number of type 2 diabetes and hyperglycaemia cases prevented by the intervention. Our estimation of health-care costs saved is very conservative, as we have not included productivity loss. Moreover, average cost of seeking care for diabetes in our study is lower than other published studies in Bangladesh,[2,3] which reported an average cost of around INT$1148.

The results for both impact of the intervention at scale and health-care costs saved are presented in Supplementary Tables 7 and 8.

---

[1] Roberts S, Craig D, Adler A, McPherson K, Greenhalgh T. Economic evaluation of type 2 diabetes prevention programmes: Markov model of low- and high-intensity lifestyle programmes and metformin in participants with different categories of intermediate hyperglycaemia. *BMC Med* 2018; **16:** 16.

[2] Sarker AR, Sultana M. Health and economic burden of diabetes in Bangladesh: priorities for attention and control. *J Diabetes* 2017; **9:** 1118-19.

[3] Islam SMS, Lechner A, Ferrari U, et al. Healthcare use and expenditure for diabetes in Bangladesh. *BMJ Glob Health* 2017; **2:** e000033.

Supplementary Methods 2: Estimating the number of DALYs averted by PLA intervention

We calculated DALYs averted using the Global Burden of Disease (GBD) study approach.[4,5] DALY is the sum of years of life lost due to premature death (YLLs) and years lived with disability (YLDs). As per the GBD approach, we used the normative standard life expectancy of 86 years. YLL saved was calculated using the following formula:

YLL saved = number of premature deaths averted by PLA*life expectancy at the age of death

We calculated the number of premature deaths averted by multiplying number of cases of diabetes and intermediate hyperglycaemia averted by PLA and death rates among these groups. Death rates for diabetes and intermediate hyperglycaemia were assumed to be twice as high as the death rate among individuals without diabetes.[6] We obtained the death rate for different age groups from the Bangladesh Life-Tables.[7] The average age of individuals with diagnosed diabetes in our end-of-study survey sample was 52.6 years, and we used this as the assumed average age of onset (or diagnosis) of diabetes in our study population. Using these assumptions and considering the Bangladeshi life expectancy at birth of 73 years,[8] we calculated the total number of premature deaths averted by PLA (Supplementary Table 9).

To calculate life expectancy at the age of death, we deducted standard life expectancy of 86 from the estimated age of death for each age group (Supplementary Table 9).

YLD saved was calculate as follows:

YLD = number of diabetes and intermediate hyperglycaemia averted by PLA*duration until death *disability weight

Duration until death was calculated for each age group by deducting age at onset of diabetes from estimated age of death for each age group. We used the GBD 2016 disability weight for uncomplicated diabetes (0.049)[9] for our calculation.

Using the approach explained above, total DALYs averted was estimated to be approximately 6800 (YLL=4300 plus YLD=2500). As a next step, we discounted the total number of DALYs averted at 3%, as recommended by WHO-CHOICE[10] and the Gates/iDSI Reference Case for Economic Evaluation.[11]

Cost per DALY averted for combined cases of diabetes and intermediate hyperglycaemia prevented was estimated to be approximately INT$124, which was calculated by dividing the total cost of PLA intervention (ie, INT$601,484) by the total discounted DALYs averted (ie, 4866).

---

[4] Murray CJL, Vos T, Lozano R, et al. Disability-adjusted life years (DALYs) for 291 diseases and injuries in 21 regions, 1990-2010: a systematic analysis for the Global Burden of Disease Study 2010. *Lancet* 2012; **380:** 2197-223.

[5] WHO. Metrics: Disability-Adjusted Life Year (DALY). Quantifying the Burden of Disease from mortality and morbidity, 2012. http://www.who.int/healthinfo/global_burden_disease/metrics_daly/en/

[6] Public Health Agency of Canada. Report from the National Diabetes Surveillance System: Diabetes in Canada, 2009. http://www.phac-aspc.gc.ca/publicat/2009/ndssdic-snsddac-09/pdf/report-2009-eng.pdf

[7] Global Health Observatory data repository. http://apps.who.int/gho/data/?theme=main&vid=60120

[8] http://data.un.org/Data.aspx?q=life+expectancy&d=PopDiv&f=variableID%3a68

[9] http://ghdx.healthdata.org/record/global-burden-disease-study-2016-gbd-2016-disability-weights

[10] Tan-Torres Edejer T BR, Adam T, Hutubessy R, Acharya A, Evans DB, Murray CJ. Making choices in health: WHO guide to cost-effectiveness analysis. Geneva: World Health Organization, 2003.

[11] laxton K, Revill P, Sculpher M, Wilkinson T, Cairns J, Briggs A. The Gates Reference Case for Economic Evaluation. Bill and Melinda Gates Foundation, 2014.

The number of DALYs averted will vary depending on life expectancy used (local life expectancy vs standard life expectancy of 86 years) and the discount rate. We plan to assess these changes as well as other uncertain parameters in future work.

Supplementary Table 1: mHealth message focus, purpose, and behaviour change techniques applied

Adapted from Jennings H, Morrison J, K A, et al. (2019) Developing a theory-driven contextually relevant mHealth intervention. *Glob Health Action* 12:1, DOI: 10.1080/16549716.2018.1550736

Focus area	Purpose of messages	Behaviour change techniques applied
General information on diabetes	Improve knowledge about the causes, symptoms and consequences of diabetes.<br><br>Challenge incorrect beliefs/misconceptions about diabetes, including perceived lack of control over one's health.<br><br>Build on knowledge and opportunities to improve health identified in formative research.	Shaping knowledge; information about health consequences; modelling behaviour.
Care seeking	Inform on where to seek blood glucose testing, advice and care for diabetes.<br><br>Inform on the importance of care seeking and medication adherence when prescribed.<br><br>Challenge fatalism and mis-conceptions about the potential to prevent and control diabetes.	Shaping knowledge; information about health consequences; modelling behaviour; encouraging social support.
Diet	Provide basic information on healthy diets and dietary considerations for people with diabetes.<br><br>Build on existing concepts of healthy and unhealthy food.<br><br>Provide information on local resources for more information and for health food.<br><br>Offer potential strategies for individuals and households to eat more healthily.	Shaping knowledge; information about health consequences; modelling behaviour; balancing pros and cons of a healthy diet; encouraging social support.
Physical activity	Inform of the role of physical activity in preventing and controlling diabetes.<br><br>Challenge mis-conceptions about who should be physically active and address perceived barriers to increasing physical activity.<br><br>Reinforce existing positive perceptions of physical activity.<br><br>Offer potential strategies for individuals and groups to increase physical activity.	Shaping knowledge; information about consequences; modelling behaviour; balancing pros and cons of physical activity; goal setting; repetition/habit formation; encouraging social support.
Smoking & tobacco use	Inform of the harmful effects of tobacco consumption on health.<br><br>Address issues of addiction and the challenges of quitting.<br><br>Provide advice on methods to quit.	Shaping knowledge; information about health consequences; goal setting; modelling behaviour; balancing pros and cons of quitting smoking.
Stress	Inform on the health consequences of stress, particularly in relation to diabetes.<br><br>Provide advice on methods of coping with stress.<br><br>Encourage emotional support of friends and relatives.	Shaping knowledge; information about health consequences; modelling behaviour; encouraging social support.

Supplementary Table 2: Glycaemic definitions and diagnostic criteria

Definitions based on: WHO, International Diabetes Federation. Definition and diagnosis of diabetes mellitus and intermediate hyperglycaemia: report of a WHO/IDF consultation. Geneva: World Health Organization, 2006.

**Definition**		**Diagnostic Criteria**
Normoglycaemia		Fasting plasma glucose <6.1mmol/L
Intermediate hyperglycaemia	Impaired fasting glucose	Fasting plasma glucose ≥6.1 mmol/L to <7.0 mmol/L<br>**AND**<br>2-h post ingestion of 75 g glucose load plasma glucose <7.8 mmol/L
	Impaired glucose tolerance	Fasting plasma glucose <7.0mmol/L<br>**AND**<br>2-h post ingestion of 75 g glucose load plasma glucose ≥7.8 mmol/L to <11.1 mmol/L
Type 2 diabetes		Fasting plasma glucose ≥7.0 mmol/L<br>**OR***<br>2-h post ingestion of 75 g glucose load plasma glucose ≥11.1 mmol/L

*Diabetes cannot be excluded without 2-h post oral glucose load test.

Supplementary Table 3: DMagic secondary outcome measures between trial arms at baseline

		Allocation		
		**Community PLA**	**mHealth**	**Control**
**Blood pressure**	**Mean diastolic blood pressure (SD)**	71.6 (11.3)	71.1 (11.2)	70.6 (11.3)
	**Mean systolic blood pressure (SD)**	121.9 (19.8)	121.2 (19.6)	120.6 (19.3)
	**Hypertension (%)**	809 (20.0%)	808 (19.9%)	764 (18.8%)
	**Hypertension control (%) (among those with known hypertension)**	138 (35.8%)	205 (42.4%)	144 (34.9%)
**Overweight & obesity**	**Mean BMI (SD)**	21.5 (3.5)	21.6 (3.6)	21.3 (3.5)
	**Overweight or obese (%)**	1241 (30.6%)	1264 (31.1%)	1145 (28.1%)
	**Abdominal obesity (%)**	1837 (46.0%)	1937 (47.9%)	1868 (46.6%)
**Quality of life & wellbeing**	**Median EQ5D* (IQR)**	0.80 (0.73-1)	0.80 (0.73-1)	0.73 (0.69-0.85)
	**Mean Self Rated Health (SD)**	73.5 (21.1)	72.3 (21.9)	72.1 (21.5)
**Psychological distress among self-reported diabetics**	**Median (IQR) SRQ20 score among adults aged 30 years and above with self-reported diabetes**	9.5 (5.0)	9.1 (5.5)	10.3 (5.3)
**Diabetes**	**Ability to report one or more valid *causes* of diabetes (%)**	1285 (31.7%)	1246 (30.6%)	1474 (36.7%)
	**Ability to report one or more valid *symptoms* of diabetes (%)**	2354 (58.5%)	2097 (51.5%)	2183 (53.9%)
	**Ability to report one or more valid *complications* of diabetes (%)**	1251 (31.1%)	999 (24.5%)	950 (23.5%)
	**Ability to recognise one or more valid *complications* of diabetes when prompted (%)**	2786 (69.3%)	2862 (66.9%)	2708 (66.9%)
	**Ability to report one or more valid ways to *prevent* diabetes (%)**	1713 (42.6%)	1385 (34.0%)	1404 (34.7%)
	**Ability to report one or more valid ways to *control* diabetes (%)**	2738 (68.1%)	2515 (61.8%)	2740 (67.7%)
	**Diabetes control (%) (among those with known diabetes)**	24 (20.9%)	18 (16.8%)	19 (22.6%)
	**Self-awareness of diabetic status among all those identified as diabetic by objective blood glucose test (n=1228) (%)**	118 (26.2%)	108 (26.7%)	84 (22.6%)
	**Receipt of professional treatment or advice for diabetes among those aware of their status (n=310) (%)**	93 (78.8%)	91 (84.3%)	72 (85.7%)
**Physical Activity**	**Average of 150 min or more doing physical activity per week (%)**	2937 (73.0%)	2871 (70.5%)	3054 (75.4%)
**Fruit & vegetable consumption**	**Mean portions of fruit and/or vegetables consumed per day (SD)**	3.6 (1.8)	3.8 (2.1)	4.0 (2.1)

Supplementary Table 4: End-of-study sociodemographic characteristics, by group

Characteristic		Control (n = 3830; 32 villages)	mHealth (n = 3817; 32 villages)	PLA (n = 3807; 32 villages)
**Age**	**30-39 years**	1266 (33.1%)	1159 (30.4%)	1225 (32.1%)
	**40-49 years**	1030 (26.9%)	1012 (26.5%)	1011 (26.6%)
	**50-59 years**	705 (18.4%)	809 (21.2%)	748 (19.7%)
	**60-69 years**	550 (14.4%)	555 (14.5%)	537 (14.1%)
	**70-100 years**	279 (7.3%)	282 (7.4%)	286 (7.5%)
**Sex**	**Male**	1801 (47.0%)	1745 (45.7%)	1741 (45.7%)
	**Female**	2029 (53.0%)	2072 (54.3%)	2066 (54.3%)
**Education**	**None**	1841 (48.6%)	1581 (41.6%)	1527 (40.3%)
	**Primary**	988 (26.1%)	1013 (26.6%)	1017 (26.8%)
	**Secondary**	947 (25.0%)	1206 (31.7%)	1250 (33.0%)
	**Tertiary**	9 (0.2%)	2 (0.1%)	0
**Illiterate**	**Literate**	1250 (33.0%)	1470 (38.7%)	1584 (41.7%)
	**Illiterate**	2536 (67.0%)	2332 (61.3%)	2211 (58.3%)
**Marital Status**	**Married**	3365 (88.9%)	3353 (88.2%)	3389 (89.3%)
	**Not married**	421 (11.1%)	449 (11.8%)	406 (10.7%)
**Religion**	**Muslim**	3441 (90.9%)	3390 (89.2%)	3443 (90.7%)
	**Other**	344 (9.1%)	412 (10.8%)	352 (9.3%)
**Occupation**	**Not working**	2128 (56.2%)	2198 (57.8%)	2168 (57.1%)
	**Manual labour**	1298 (34.3%)	1264 (33.3%)	1249 (32.9%)
	**Non-manual labour**	360 (9.5%)	340 (8.9%)	377 (9.9%)
**Wealth quintile**	**Most poor**	649 (17.1%)	675 (17.8%)	935 (24.6%)
	**Very poor**	733 (19.4%)	846 (22.3%)	892 (23.5%)
	**Poor**	750 (19.8%)	720 (18.9%)	680 (17.9%)
	**Less poor**	813 (21.5%)	832 (21.9%)	642 (16.9%)
	**Least poor**	841 (22.2%)	729 (19.2%)	646 (17.0%)

Data on education, literacy, marital status, religion, occupation, and wealth only gathered from survey participants (n= 11,383). Missing education data for 2 participants (1 community arm, 1 control arm), missing occupation data for 1 participant in community arm, and missing religion for 1 participant in control arm.

Supplementary Table 5: Prespecified sensitivity analysis

	Arm	Model adjustment			
		(a) accounting for stratified, clustered design	(b) a + plus adjustment for wealth	(c) b + adjusted for inclusion in baseline survey	(d) a + multilevel multiple imputation for missing blood glucose measures
		OR (95% CI)	OR (95% CI)	OR (95% CI)	OR (95% CI)
**Primary Outcome 1: Prevalence of diabetes & intermediate hyperglycaemia**	**Control**	Ref	Ref	Ref	Ref
	**Community groups**	0.36 (0.27, 0.48); p<0.0001	0.35 (0.26, 0.47); p<0.0001	0.36 (0.27, 0.47); p<0.0001	0.37 (0.27, 0.49) p<0.0001
	**mHealth**	0.94 (0.75, 1.18); p=0.605	0.93 (0.74, 1.16); p=0.504	0.93 (0.74, 1.16) p=0.525	0.93 (0.74, 1.18) p=0.563
**Primary Outcome 2: Diabetes among baseline intermediate hyperglycaemia cohort**	**Control**	Ref	Ref	Ref	Ref
	**Community groups**	0.39 (0.24, 0.65); p=0.0005	0.38 (0.23, 0.63); p=0.0003	Na	*0.39 (0.25, 0.62) p=0.0001
	**mHealth**	0.96 (0.68, 1.35); p=0.794	0.97 (0.70, 1.39); p=0.937	Na	*0.92 (0.67, 1.27) p=0.622

^Multiple imputation based on age, sex, intervention allocation, and cluster.

*Multiple imputation based on baseline measurements of: sex, age, wealth quintile, smoking status, BMI, hypertension, fasting and 2-hour blood glucose reading, and intervention allocation. Diabetes outcome among the intermediate hyperglycaemia cohort did not vary in some clusters, so clustering was not included in the multiple imputation model in this case.

Supplementary Table 6: Post-hoc sensitivity analysis of PLA community intervention effects on continuous blood glucose measurements

	Arm	Mean (SD)	Regression model adjustment			
			(a) accounting for stratified, clustered design	(b) a + plus adjustment for wealth	(c) a + enumerator fixed effects	
			Coef. (95% CI)	Coef. (95% CI)	Coef. (95% CI)	Enumerator F-statistic (p-value)
**Mean fasting glucose**	**Control**	5.85 (1.17)	Ref	Ref	Ref	F=0.002 (p=0.9642)
	**Community groups**	5.57 (0.83)	-0.29 (-0.39, -0.19); p<0.0001	-0.30 (-0.40, -0.20); p<0.0001	-0.29 (-0.39, -0.19); p<0.0001	
**Mean 2-hour glucose**	**Control**	7.58 (2.37)	Ref	Ref	Ref	F=0.966 (p=0.3257)
	**Community groups**	6.99 (1.67)	-0.59 (-0.80, -0.37); p<0.0001	-0.61 (-0.82, -0.40); p<0.0001	-0.59 (-0.81, -0.37); p<0.0001	

Supplementary Table 7: Impact of PLA community mobilisation at scale with no loss of efficacy

2016 Incidence rate for diabetes (GBD 2016)	Incidence (Number)	Incidence for both diabetes & intermediate hyperglycaemia (Number)	PLA community mobilisation RR (with no loss of efficacy)	Number of Diabetes & intermediate hyperglycaemia with D-magic scale up	Cases averted with D-magic	% averted	Cost per case averted	Annual health care cost saving (INT$)
268	442,136	884,272	0.61	539,406	344,866	39%	546	188,207,477

Supplementary Table 8: Impact of PLA community mobilisation at scale with 30% loss of efficacy

2016 Incidence rate for diabetes (GBD 2016)	Incidence (Number)	Incidence for both diabetes & hyperglycaemia (Number)	D-Magic RR (with 30% loss of efficacy)	Number of Diabetes & intermediate hyperglycaemia with D-magic scale up	Cases averted with D-magic	% averted	Cost per case averted	Annual health care cost saving (INT$)
268	442,136	884,272	0.73	642,865	241,406	27%	546	131,745,234

Supplementary Table 9: Estimated total DALYs averted by PLA intervention

Age group	Death rate (Bangladesh Life-Table)	Death rate for diabetes (Death rate*2)	Total deaths	Diabetes related deaths (0.5*total deaths)	Total individuals alive	Age at death	Duration until death	YLD	Life expectancy at the age of death	YLL
					1904*					
50-54 years	0.008	0.016	30	15	1874	52	0	0	34	518
55-59 years	0.011	0.022	41	21	1832	57	4	4	29	598
60-64 years	0.023	0.046	84	42	1748	62	9	19	24	1011
65-69 years	0.027	0.054	94	47	1654	67	14	33	19	897
70-74 years	0.055	0.11	182	91	1472	72	20	2408	14	1273
Total			432	216				2465		4297
**DALYs averted (0% discounted)**										**6762**

* Total number of diabetes and intermediate hyperglycaemia averted by PLA (calculated as the difference between the expected and the actual number of cases using the adjusted odds ratio relative to the control population).[12]

[12] Haghparast-Bidgoli H, Shaha SK, Kuddus A, et al. Protocol of economic evaluation and equity impact analysis of mHealth and community groups for prevention and control of diabetes in rural Bangladesh in a three-arm cluster randomised controlled trial. *BMJ Open* 2018; **8:** e022035.
